# Use of ACEi/ARBs, SGLT2 inhibitors and MRAs can help us reach the therapeutic ceiling in CKD

**DOI:** 10.1093/ckj/sfae014

**Published:** 2024-01-31

**Authors:** Pantelis Sarafidis

**Affiliations:** 1^st^ Department of Nephrology, Aristotle University of Thessaloniki, Hippokration Hospital, Thessaloniki, Greece

**Keywords:** chronic kidney disease, mineralocorticoid receptor antagonists, sodium–glucose cotransporter inhibitors, type 2 diabetes

## Abstract

Chronic kidney disease (CKD) is increasing in prevalence worldwide, posing major implications for public health such as kidney failure requiring dialysis, and increased risk of cardiovascular and all-cause mortality. Diabetic and hypertensive kidney disease represent the two most common causes of CKD. Until a few years ago, lifestyle modifications, blood pressure, glycaemic and lipid control, along with angiotensin-converting enzyme inhibitor or angiotensin-receptor blocker monotherapy were the only measures for retarding these two diseases and were the cornerstone of treatment for CKD of any aetiology. Effective application of all these measures could reduce the estimated glomerular filtration rate (eGFR) decline in proteinuric CKD roughly from 10–12 to 5–6 mL/min/1.73 m^2^/year, hence leaving a large unmet need in CKD treatment. In recent years, major kidney outcome trials showed that the addition of sodium–glucose cotransporter-2 inhibitors (SGLT2i) in patients with CKD with or without type 2 diabetes (T2D) and of the non-steroidal mineralocorticoid receptor antagonist finerenone in patients with CKD with T2D can largely improve kidney and cardiovascular outcomes. Elegant analyses of these trials shed further light on these effects, showing that SGLT2i or finerenone use on top of standard-of-care treatment in patients with albuminuric CKD can further reduce chronic eGFR annual loss to 2–2.5 mL/min/1.73 m^2^, while SGLT2is in normoalbuminuric CKD can reduce this loss <0.5 mL/min/1.73 m^2^, i.e. well below the aging-related GFR loss. Therefore, current evidence suggests that available treatments, if properly implemented, can help us reach the therapeutic ceiling in the majority of CKD patients.

## INTRODUCTION

Chronic kidney disease (CKD) is defined as abnormalities of kidney function or structure that are present for >3 months, with most common diagnostic criteria being estimated glomerular filtration rate (eGFR) <60 mL/min/1.73 m^2^ or urinary albumin to creatinine ratio (UACR) >30 mg/g [1]. This disease has major implications for public health, including an increased risk of CKD progression to kidney failure requiring kidney replacement therapy and of acute kidney injury (AKI) of various aetiologies, as well as of premature cardiovascular and all-cause mortality [2]. Recent estimates suggest that around 850 million people worldwide have CKD, with 3.9 million receiving kidney replacement therapy [3], while according to valid projections CKD is expected to become the fifth global cause of death by the year 2040 [4].

Diabetic kidney disease (DKD) is perhaps the largest contributor to these adverse projections for CKD. It develops in up to 40% of patients with diabetes mellitus, while following a continuous increase in the prevalence of type 2 diabetes (T2D) over the past decades, it was estimated that approximately 460 million people were living with diabetes in 2019; the prevalence is predicted to rise to 700 million by 2045 [5, 6]. Hypertensive kidney disease *per se* is the second most important cause of kidney failure in most countries [7, 8], while elevated blood pressure (BP) is the most common modifiable risk factor for the progression of CKD of any aetiology [9, 10]. Similar to diabetes, all recent estimates suggest a continuous increase in the prevalence of hypertension worldwide moving from roughly 1.1 billion people in 2015 to 1.4 billion in 2050, with increases in the rates of uncontrolled and resistant hypertension [11–13], all of which are expected to affect, among other things, the incidence and progression of CKD.

The association of CKD with cardiovascular events and mortality is well established [14], as both increase exponentially with decreasing eGFR or increasing albuminuria independent of age, sex and other risk factors [15, 16]. It is also known that for patients with CKD stage 3 the risk of cardiovascular death is at least 10 times higher than the risk of kidney failure [17]. Furthermore, large observational studies reported the presence of a morbid interaction when both CKD and diabetes are present; the 10-year cumulative cardiovascular mortality for patients without diabetes or CKD, with diabetes and no CKD, and with CKD and no diabetes was estimated at 3.4%, 6.7% and 9.9%, respectively, but for those with both diabetes and CKD it increased to 19.6% [18].

Following the above, it is more than clear that delaying the onset and progression of CKD must be a major goal for public health worldwide. Towards this, lifestyle modifications, BP, glycaemic and lipid control, along with the use of an angiotensin-converting enzyme inhibitor (ACEi) or an angiotensin receptor blocker (ARB), have been the cornerstone of CKD treatment for decades [1, 19, 20]. In recent years, several studies with hard kidney outcomes have shown important nephro- and cardioprotection with sodium–glucose cotransporter-2 inhibitors (SGLT2is) in patients with CKD with or without T2D and with the non-steroidal mineralocorticoid receptor antagonist (MRA) finerenone in patients with CKD with T2D, leading to recommendations of using these agents as first-line treatment in the relevant patient groups [20–24].

## USE OF AN ACEi OR AN ARB TO DELAY CKD PROGRESSION: THE CORNERSTONE OF TREATMENT

Several research efforts, including trials with hard kidney outcomes, of the past decades provided sufficient evidence that single renin–angiotensin system (RAS) blockade with an ACEi or an ARB can slow down the progression of albuminuric diabetic or non-diabetic CKD [19]. The Reduction-of-Endpoints-in-NIDDM-with-the-Angiotensin-II-Antagonist-Losartan (RENAAL) study randomized 1513 participants with T2D and overt nephropathy to receive treatment with losartan or placebo [25]. Losartan was associated with 16% reduction in doubling of serum creatinine (SCr), end-stage renal disease (ESRD) or death, 35% reduction in ACR and 15% decrease in the rate of creatinine clearance reduction. In the Irbesartan-Diabetic-Nephropathy-Treatment (IDNT) study, 1715 hypertensive patients with T2D and overt nephropathy were randomized to irbesartan, amlodipine or placebo [26]. Irbesartan resulted in 20% reduction compared with placebo and 23% reduction compared with amlodipine in the risk of doubling of SCr, ESRD or death; proteinuria decreased by 33% with irbesartan versus 6% with amlodipine, and 10% with placebo. In the Ramipril-Efficacy-In-Nephropathy (REIN) trial, which randomized 352 patients with non-diabetic proteinuric CKD in ramipril or placebo, ramipril was associated with a lower rate of eGFR decrease per month and a greater reduction in proteinuria when compared with placebo, independently of the antihypertensive effect [27]. Finally, in the African-American-Study-on-Kidney-Disease (AASK), which assigned 1094 African-American individuals to receive ramipril or metoprolol or amlodipine, ramipril offered 22% and 38% reductions compared with metoprolol and amlodipine, respectively, in the risk of the primary endpoint (reduction of GFR ≥50%, ESRD or death), along with relevant differences in proteinuria [28]. A set of elegant *post hoc* analyses of these trials confirmed the major haemodynamic pathway through which both ACEis and ARBs confer nephroprotection, as they produce an eGFR ‘dip’ of around 10%–15% within the first weeks of treatment, corresponding to decrease in glomerular pressure and per nephron hyperfiltration, and suggested that the nephroprotective effect of these agents was directly proportionate to the magnitude of proteinuria/albuminuria reduction over the first months of treatment [19, 29–31].

The above solid evidence for the nephroprotection offered by ACEis and ARBs is mainly related to patients with CKD and proteinuria/severely increased albuminuria (UACR >300 mg/g). For patients with moderately increased albuminuria (UACR 30–300 mg/g) a similar effect is suggested; herein the evidence is less strong, as relevant trials have examined intermediate outcomes (i.e. UACR change or progression to higher UACR category) [32], but yet convincing enough for major guideline documents to recommend an ACEi or an ARB for nephroprotection in all CKD patients with moderately or severely increased albuminuria [1, 22]. However, in patients with normoalbuminuric CKD, which represent lately an important proportion of the CKD population, there is no hard evidence that ACEis or ARBs offer additional nephroprotection to that expected from BP lowering [19, 33]. For this category of patients, the indication for using ACEis or ARBs is hypertension rather than nephroprotection, and the relevant evidence derives from outcome trials in hypertensive patients that rendered these drug classes first-line choices for hypertension treatment as monotherapy or in combination regimens [20, 34, 35].

Several additional points need to be considered regarding RAS blockade treatment. First, that the two major renal outcome trials studying the effects of double RAS blockade on renal and cardiovascular outcomes in proteinuric DKD, i.e. the Aliskiren-Trial-in-Type-2-Diabetes-Using-Cardiorenal-Endpoints (ALTITUDE) evaluating aliskiren versus placebo on top of ACEi or ARB treatment [36] and The Veterans-Affairs-Nephropathy-in-Diabetes (VA NEPHRON-D) study that aimed to compare a combination of losartan and lisinopril versus losartan alone, were prematurely terminated due to increased rates of hyperkalemia and AKI with no differences between arms in the primary or secondary renal endpoints [37]. Following these results, the combined use of ACEis with ARBs or aliskiren was suggested to be contraindicated in all populations [1, 20, 22] and was abandoned in clinical practice. Second, in an ideal scenario combined with glycaemic and BP control, single RAS blockade may reduce the rate of eGFR decline in proteinuric CKD from about 10–12 to 5–6 mL/min/1.73 m^2^/year [38, 39]. Despite this being great progress, it signifies a quite high residual renal risk, since in healthy individuals this decline is estimated at around 1–1.5 mL/min/1.73 m^2^ per year [5, 39]. Third, several factors may interfere with optimal use of single RAS blockade in everyday clinical practice, including the risk of AKI and hyperkalemia [40, 41]. Finally, and perhaps more importantly, in all the aforementioned landmark renal trials with single RAS blockade in proteinuric CKD, no differences in cardiovascular outcomes or all-cause mortality were evident between treatment groups [42]. Although it could be argued that this could be related to sample sizes being insufficient to detect differences in these outcomes, systematic reviews and meta-analyses reaching sample sizes up to >20 000 individuals also confirmed the absence of beneficial effects of ACEis and ARBs compared with placebo or active treatment on cardiovascular outcomes and all-cause mortality in patients with CKD [43–45]. Such findings rather suggest that ACEis and ARBs, although offering nephroprotection, are not associated with reduction in cardiovascular disease in this population [42].

## SGLT2is: GOOD NEWS COMING AS A SURPRISE

After almost two decades without major breakthroughs in nephrology, and with several promising options (double RAS blockade, thiazolidinediones, steroidal MRAs, nuclear factor κB pathway inhibitors, endothelin-1 receptor antagonists and others) failing to provide hard evidence on efficacy and safety for retarding CKD progression, the first hints about potential nephroprotection with SGLT2is, a class of oral hypoglycaemic agents (documented with albuminuria reduction in T2D) came as a surprise and encountered some skepticism [46, 47]. Soon, however, renal outcome data from large cardiovascular trials with SGLT2is provided convincing answers. The Empagliflozin-Cardiovascular-Outcome-Event-Trial-in-Type-2-Diabetes-Mellitus-Patients (EMPA-REG OUTCOME) trial randomized 7028 patients with T2D to 10 or 25 mg of empagliflozin versus placebo; more than 3600 patients had increased albuminuria and more than 1800 an eGFR <60 mL/min/1.73 m^2^. Apart from reductions in cardiovascular events and all-cause mortality, empagliflozin reduced the pre-specified [hazard ratio (HR) 0.61, 95% confidence interval (CI) 0.53–0.70] and the *post hoc* renal composite of doubling of SCr, initiation of renal replacement therapy or death from renal disease (HR 0.54, 95% CI 0.40–0.75) [48]. Reductions of the same magnitude in the composite renal outcomes were noted in the Canagliflozin-Cardiovascular-Assessment-Study (CANVAS) and the Multicenter-Trial-to-Evaluate-the-Effect-of-Dapagliflozin-on-the-Incidence-of-Cardiovascular-Events-Thrombolysis-In-Myocardial-Infarction 58 (DECLARE-TIMI 58) studies, respectively [49, 50], despite the different comorbidity profile of the populations and the slightly different cardiovascular effects reported in these studies.

Following the above, three studies in patients with CKD and primary renal outcomes reported their results. The Canagliflozin-and-Renal-Events-in-Diabetes-with-Established-Nephropathy-Clinical-Evaluation (CREDENCE) study [51] randomized 4401 patients with T2D, CKD and UACR from 300 to 5000 mg/g already treated with ACEis or ARBs in canagliflozin or placebo and was prematurely terminated (median follow-up 2.62 versus a projected duration of 5.5 years) due to clear benefit of canagliflozin. The relative risk of the primary outcome (ESKD defined as dialysis, transplantation, or sustained eGFR <15 mL/min/1.73 m^2^, doubling of SCr, or death from renal or cardiovascular causes) was 30% lower with canagliflozin versus placebo (HR 0.70, 95% CI 0.59–0.82), with event rates of 43.2 and 61.2 per 1000 patient-years, respectively. The relative risk of the renal-specific composite of ESKD, doubling of SCr or death from renal causes was lower by 34%, and ESKD was lower by 32% [51].

The Study-to-Evaluate-the-Effect-of-Dapagliflozin-on-Renal-Outcomes-and-Cardiovascular-Mortality-in-Patients-With-Chronic-Kidney-Disease (DAPA-CKD) randomized 4304 participants with an eGFR ≥25 and ≤75 mL/min/1.73 m^2^, and a UACR ≥200 and ≤5000 mg/g, to dapagliflozin versus placebo on top of an ACEi or ARB. Two thirds of patients had T2D, while the rest had CKD of various causes (including ischaemic/hypertensive nephropathy (16%) and chronic glomerulonephritis (16.1%)). DAPA-CKD was also prematurely terminated (median of 2.4 years) due to benefit [52]. The primary endpoint (≥50% sustained decline in eGFR, ESKD, or cardiovascular or renal death) occurred in 9.2% versus 14.5% of the participants in the dapagliflozin and the placebo groups, respectively (HR 0.61, 95% CI 0.51–0.72), a reduction apparent in all subgroups of underlying disease, while incident ESKD was also reduced by 36%. Of importance, despite the premature terminations of both CREDENCE and DAPA-CKD several cardiovascular outcomes, as well as all-cause mortality (HR 0.83, 95% CI 0.68–1.02 and HR 0.69, 95% CI 0.53–0.88, respectively), were also nominally or significantly reduced, an effect that has never appeared before in any trial in CKD [42].

EMPA-KIDNEY is the most recent renal outcome trial and significantly expanded our knowledge in the field, as it included a wider population of CKD patients. It randomized 6609 patients with CKD of various aetiologies and eGFR ≥20 but <45 mL/min/1.73 m^2^, or eGFR ≥45 but <90 mL/min/1.73 m^2^ with simultaneous UACR >200 mg/g, to empagliflozin 10 mg or placebo [53]. The study was also stopped prematurely due to benefit, showing during a median follow-up of 2.0 years significant reductions with empagliflozin in the risk of progression of kidney disease (ESKD, decrease in eGFR to <10 mL/min/1.73 m^2^, decrease in eGFR of ≥40% from baseline or death from renal causes) or death from cardiovascular causes (HR 0.72, 95% CI 0.64–0.82). Results were consistent among patients with or without diabetes and across subgroups defined according to eGFR ranges.

## NON-STEROIDAL MRAs: MORE GOOD NEWS THAT WERE LONG AWAITED

Steroidal MRAs (spironolactone and eplerenone) have been used in clinical practice for many years for the treatment of primary aldosteronism, resistant hypertension and heart failure with reduced ejection fraction [54]. With solid evidence on various mechanisms through which MR activation can produce kidney injury and documentation of aldosterone escape during ACEi and ARB monotherapy [54–56], several trials examined and showed that steroidal MRAs effectively reduce albuminuria in patients with diabetic and non-diabetic CKD [57]; however, their use was curtailed due to lack of trials with hard outcomes and the potential risks of hyperkalemia [56]. In recent years, novel nonsteroidal MRAs have been developed, among which one was recently associated in two outcome trials with significant reductions in renal and cardiovascular outcomes in patients with T2D and moderately or severely increased albuminuria [58, 59]. FIDELIO-DKD (Finerenone-in-reducing-kidney-failure-and-disease-progression-in-Diabetic-Kidney-Disease) randomized 5734 T2D patients with either UACR 30–<300 mg/g, eGFR 25–<60 mL/min/1.73 m^2^ and diabetic retinopathy or UACR 300–5000 mg/g and eGFR 25 to <75 mL/min/1.73 m^2^ to finerenone 10–20 mg or placebo on top of a maximum tolerated RAS blocker monotherapy [58]. During a median of 2.6 years of follow-up, finerenone significantly reduced the primary outcome (kidney failure, i.e. dialysis for ≥90 days or kidney transplantation or eGFR <15 mL/min/1.73 m^2^, sustained (≥4 weeks) decrease in the eGFR ≥40% from baseline, or death from renal causes) compared with placebo (HR 0.82, 95% CI 0.73–0.93). This was also the case for the secondary cardiovascular outcome (HR 0.86, 95% CI 0.75–0.99). Hyperkalemia episodes leading to drug discontinuation were more frequent with finerenone compared with placebo but were relatively uncommon (2.3% vs 0.9%). FIGARO-DKD (Finerenone-in-reducing-cardiovascular-mortality-and-morbidity-in-Diabetic-Kidney-Disease) included 7437 patients with T2D and either UACR ≥30–<300 mg/g and eGFR ≥25–90 mL/min/1.73 m^2^ or UACR ≥300–≤5000 mg/g and eGFR ≥60 mL/min/1.73 m^2^, i.e. a population with relatively lower risk for CKD progression [59]. During a median follow-up of 3.4 years, the primary outcome (cardiovascular death, nonfatal MI, nonfatal stroke or hypertensive heart failure) was reduced with finerenone (HR 0.87, 95% CI 0.76–0.98). Finerenone was also associated with a marginally significant lower risk for the key secondary renal outcome (kidney failure, sustained decrease in the eGFR ≥40% from baseline or renal death; HR 0.87, 95% CI 0.76–1.01) and a significant reduction in incidence of ESKD (HR 0.64, 95% CI 0.41–0.995).

It is worth noting that these reductions in the risk of hard renal outcomes with finerenone were originally seen as numerically smaller than those observed with SGLT2is in relevant major trials [51–53]. However, this appears to be largely related to the actual population under study, as exemplified by a *post hoc* analysis of the FIDELIO-DKD [60] including only patients who met the CKD inclusion criteria of the CREDENCE study (UACR >300–5000 mg/g and eGFR 30–<90 mL/min/1.73 m^2^) and showing reductions of similar magnitude with canagliflozin in kidney and cardiovascular endpoints examined. The FIDELITY analysis was a pre-specified pooled analysis of both FIDELIO-DKD and FIGARO-DKD including a total of 13 171 patients, with mean eGFR 57.6 mL/min/1.73 m^2^ and median UACR 515 mg/g; among several interesting analyses the influence of concomitant SGLT2i use on the study outcomes was examined [61]. About 6.7% of patients were on SGLT2is at baseline and 8.5% initiated one during the trials. The HRs with finerenone versus placebo for the kidney composite outcome were 0.80 (95% CI 0.69–0.92) without and 0.42 (95% CI 0.16–1.08) with SGLT2i. For the cardiovascular composite, the HRs were 0.87 (95% CI 0.79–0.96) without and 0.67 (95% CI 0.42–1.07) with SGLT2i. Patients receiving an SGLT2i at baseline had lower incidence of hyperkalemia in both the placebo and finerenone groups. These findings may suggest that an MRA and an SGLT2i may be complementary to each other in improving efficacy, as assessed by the reduction in the risk of kidney events and cardiovascular events while improving safety by reducing the risk of hyperkalemia [24].

## NEPHROPROTECTION IS AT ITS BEST WITH SGLT2is AND FINERENONE: INSIGHTS FROM eGFR SLOPE ANALYSES

The wealth of evidence deriving from recent outcome trials with SGLT2is and finerenone has improved our understanding around progression of diabetic and non-diabetic CKD. Among the various types of sub-analyses published, perhaps the most fruitful insights can be offered by analyses of eGFR loss over time with relevant treatments. Such analyses are particularly important for subgroups of patients at low risk for CKD progression (i.e. those with relatively high eGFR or normoalbuminuria), which are not expected to offer sufficient numbers of hard endpoints (i.e. progression to ESKD) to detect significant differences within the relatively short timeframe of a clinical trial, but still contribute a high percentage of individuals progressing to ESKD in real-world situations.

As has been known for several years for ACEis and ARBs [38], the typical pattern of eGFR changes suggestive of nephroprotection involves an initial eGFR drop of around 10%–15% (and up to 30%) due to haemodynamic effects over the first few weeks of treatment, followed by better preservation of eGFR over the chronic phase and reversal of the initial, functional, drop in case of discontinuation of the nephroprotective agent. Such an effect was observed in all major SGLT2i and finerenone trials described above in the overall and in subgroup populations [24, 56, 63]. For example, in the EMPA-REG OUTCOME study (including a T2D population with average eGFR at 74 mL/min/1.73 m^2^ and various degrees of albuminuria), from baseline to Week 4, weekly decreases of –0.62 ± 0.04 and –0.82 ± 0.04 mL/min/1.73 m^2^ with empagliflozin 10 and 25 mg, respectively, compared with 0.01 ± 0.04 mL/min/1.73 m^2^ (*P *< .001) with placebo were noted [48]. However, from Week 4 to treatment end, eGFR stabilized in both empagliflozin groups (annual decreases of –0.19 ± 0.11 mL/min/1.73 m^2^), while it declined steadily in patients receiving placebo on top of best available therapy with ACEis or ARBs (–1.67 ± 0.13 mL/min/1.73 m^2^, *P *< .001). After cessation of the study drug (last week of treatment to end of follow-up), eGFR increased in patients previously treated with empagliflozin (weekly increases of 0.48 ± 0.04 and 0.55 ± 0.04 mL/min/1.73 m^2^ in empagliflozin groups vs –0.04 ± 0.04 mL/min/1.73 m^2^ with placebo, *P *< .001), offering convincing evidence of the haemodynamic effects of these drugs. Of greater importance is that empagliflozin was able to significantly reduce chronic eGFR loss across all albuminuria categories, i.e. in patients with large differences in the risk of CKD progression, with the absolute benefit being obviously larger in patients with severely increased albuminuria [62]. Similar findings were observed in CANVAS and DECLARE-TIMI trials [49, 64].

**Figure 1:**
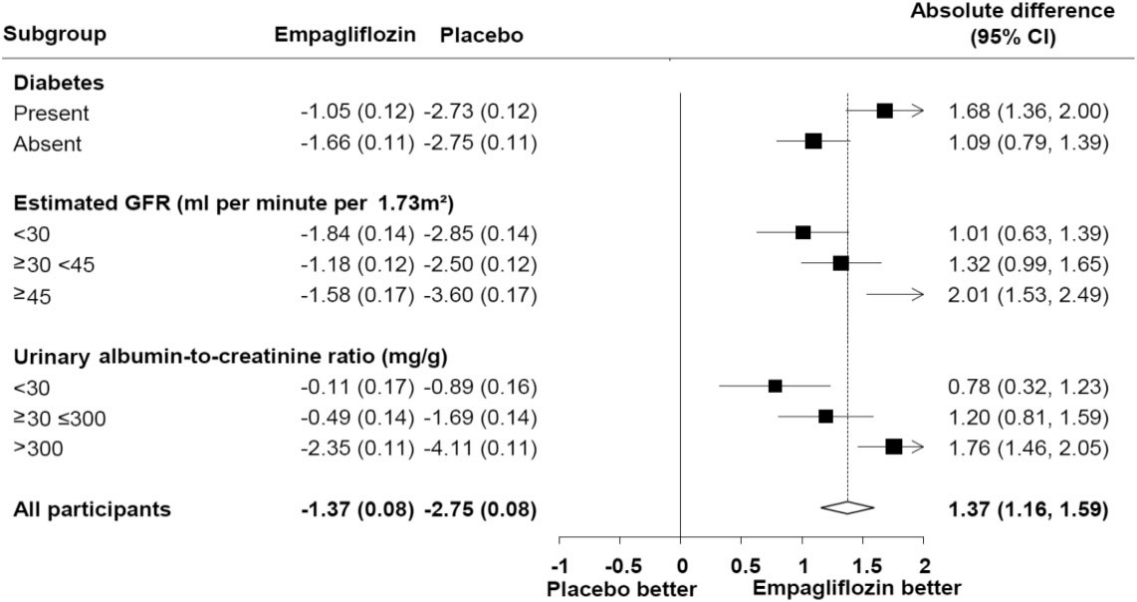
Mean annual rates of change in eGFR from 2 months to the final follow-up visit (chronic slopes) in EMPA-KIDNEY trial by key subgroups estimated using shared parameter models. From Herrington *et al*. [53], with permission.

**Figure 2:**
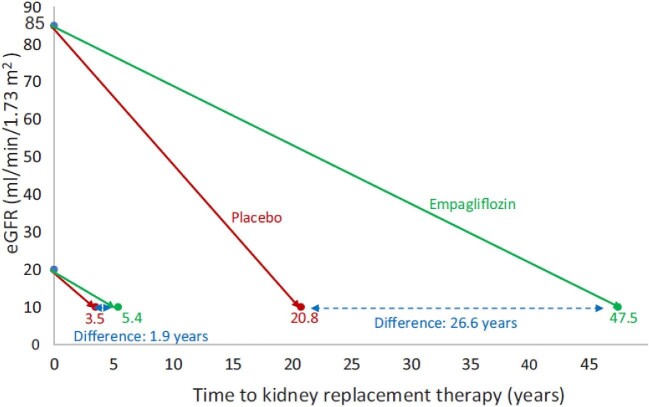
Hypothetical real-life application of chronic eGFR slopes observed in the EMPA-KIDNEY trial subgroups into time to kidney failure (defined as eGFR 10 mL/min/1.73 m^2^) for two patients at different eGFR starting points. Delay in time (years) to kidney failure on empagliflozin vs placebo, according to baseline eGFR, obtained by subtracting the time to kidney failure on empagliflozin from the time to kidney failure on placebo. From Fernández-Fernandez *et al*. [65] with permission.

Following two SGLT2i trials in proteinuric CKD with similar findings [51, 52], the EMPA-KIDNEY study has important additional insights to offer, since it purposely included a wider population of CKD individuals, with 35% of the final population having eGFR <30 mL/min/1.73 m^2^, 20% UACR 30–300 mg/g and 28% UACR <300 mg/g. As the empagliflozin-related benefit was driven by differences in the high-risk subgroups, the premature termination of the study has limited the power to observe differences in hard outcomes for low-risk subgroups [65]. However, the analyses of chronic eGFR slopes showed impressive data on empagliflozin slowing CKD progression across all eGFR and UACR categories and in patients with and without diabetes mellitus (Fig. 1) [53]. In patients with UACR >300 mg/g, empagliflozin- and placebo-related annual eGFR reductions were –2.35 vs –4.11 mL/min/1.73 m^2^, respectively. Furthermore, in participants in the low-risk subgroup of UACR <30 mg/g, the relevant numbers were –0.11 vs –0.89 mL/min/1.73 m^2^, respectively, with empagliflozin and placebo [53], i.e. SGLT2i use was associated with an 8-fold slower CKD progression. A hypothetical scenario of long-term application of the chronic eGFR loss rates in different eGFR subgroups at the EMPA-KIDNEY data suggests that addition of these agents in people of middle age with eGFR around 80–90 mL/min/1.73 m^2^ could possibly delay the risk of progression of CKD to ESKD by more than 25 years (Fig. 2) [65].

In addition to the above, in both FIDELIO-DKD and FIGARO-DKD an early dip in eGFR followed by a lower rate of eGFR loss and an early reduction in UACR by 40% which persisted over time were noted in the finerenone arms [58, 59]. In FIDELIO-DKD, annualized eGFR changes with finerenone and placebo were –2.66 vs –3.97 mL/min/1.73 m^2^, respectively (Table 1), which are practically similar to the aforementioned differences observed in the relevant patient subgroup of UACR >300 mg/g in the EMPA-KIDNEY trial [53]. This renoprotective effect of finerenone was evident even in the subgroup of patients with Stage 4 proteinuric DKD [66]. Finally, in the FIDELITY analysis by use of SGLT2is the annualized differences for patients using an SGLT2i at baseline were –1.92 vs –3.45 mL/min/1.73 m^2^ for finerenone and placebo, respectively, whereas for those without SGLT2i were –2.54 vs –3.52 mL/min/1.73 m^2^, respectively [61], indicating that use of SGLT2i and finerenone on top of ACEi or ARB can provide the better preservation of kidney function.

**Table 1:** Annualized least square mean change in eGFR from 4 months to the final follow-up visit (chronic slopes) in FIGARO-DKD, FIDELIO-DKD and relevant *post hoc* analyses.

Study/analysis	*N*	Baseline eGFR (mean) mL/min/1.73 m^2^	Baseline UACR (median)	Finerenone, annualized eGFR change (mL/min/1.73 m^2^)		Placebo, annualized eGFR change (mL/min/1.73 m^2^)	
FIDELIO-DKD [58]	5734	44.3	852 mg/g	–2.66		–3.97	
FIGARO-DKD [59]	7437	67.8	308 mg/g	–2.37		–3.50	
FIDELITY—whole population [67]	13 026	57.6	515 mg/g	–2.5		–3.7	
FIDELITY—Stage 4 CKD [66]	890	26.9	720 mg/g	–1.8		–3.2	
FIDELITY—analysis by SGLT2i use [61]	13 026	57.6	515 mg/g	With SGLT2i: –1.92	Without SGLT2i: –2.54	With SGLT2i: –3.45	Without SGLT2i: –3.72

## CONCLUSIONS

Recent evidence from major kidney outcome trials has greatly expanded our knowledge on the benefits of treatment of the most common causes of CKD with SGLTis and of diabetic CKD with SGLT2is and finerenone. Detailed post hoc analyses have also delineated the anticipated benefits with modern treatments for different subgroups of CKD patients. As such, SGLT2i use in patients with severely increased albuminuria can further reduce chronic eGFR annual loss from 4–6 mL/min/1.73 m^2^ when all previously available measures including ACEi or ARB monotherapy are optimally implemented, down to 2–2.5 mL/min/1.73 m^2^, with proportionate benefits of magnitude in patients with moderately increased albuminuria [38, 53, 62]. Finerenone added to ACEi or ARB monotherapy appeared to display similar nephroprotective properties for these two subgroups of UACR in diabetic CKD [58, 59], while the combinations of all three classes may be able to offer additional marginal benefits and reduce the eGFR loss very close to what is considered as ‘normal’ loss due to aging, i.e. around 1–1.5 mL/min/1.73 m^2^/year [61]. In individuals with normoalbuminuric CKD, data from the EMPA-KIDNEY trial suggest that eGFR loss can go down to 0.1 mL/min/1.73 m^2^/year with SGLT2i treatment, i.e. that these agents can practically ‘freeze’ CKD progression in this subgroup of patients. As such, one can reasonably argue that available treatments can help us reach the therapeutic ceiling in the majority of CKD patients who do not have immune-related disease. For this to happen, health strategies involving physician and patient education and aiming at early detection, multidisciplinary care and appropriate treatment of CKD are still urgently needed worldwide.
